# Trends in Drug-Related Mortality in Adults Aged 40–59 in the United States (2018–2023): A Proxy Analysis of Polypharmacy Using CDC WONDER (Wide-Ranging Online Data for Epidemiologic Research)

**DOI:** 10.7759/cureus.95797

**Published:** 2025-10-31

**Authors:** Laraib, Uzma Khalid, Ayesha Khalid

**Affiliations:** 1 General Medicine, Quaid-E-Azam Medical College, Bahawalpur, PAK; 2 Medicine and Surgery, Combined Military Hospital (CMH) Lahore Medical College and Institute of Dentistry, Lahore, PAK; 3 Medicine, University College of Medicine and Dentistry, Lahore, PAK

**Keywords:** drug related mortality, epidemiology, polypharmacy, public health, united states

## Abstract

Introduction: Drug-related mortality is a crucial public health issue in the United States, especially among middle-aged adults, in whom polypharmacy raises the likelihood of adverse events. This study aims to explore the trends in drug-related deaths among adults aged 40-59 from 2018 to 2023 in the United States, using mortality as an exploratory proxy to investigate the broader implications of polypharmacy.

Methods: A cross-sectional analysis was conducted using International Classification of Diseases, Tenth Revision (ICD-10)-specific codes from CDC WONDER (Wide-ranging ONline Data for Epidemiologic Research). For the years 2018-2023, crude mortality rates were evaluated for middle-aged individuals aged 40-59, classified into five age groups. Yearly trends were assessed to evaluate changes over time.

Results: The age group 50-54 consistently demonstrated an elevated crude mortality rate, peaking in 2020. On the contrary, the 40-44 and 55-59 age groups showed lower rates with slight variations, particularly a visible increase was seen in 2020 among most age groups, coinciding with the COVID-19 pandemic, followed by stabilization in the subsequent years.

Conclusions: Drug-related mortality in the United States is a significant issue among middle-aged adults, with the 50-54 age group displaying the highest rates. The noticeable rise in 2020 may indicate pandemic-related aspects, including healthcare accessibility, and a more psychosocial burden. These findings underline the need for focused measures to alleviate polypharmacy risks in this population.

## Introduction

Polypharmacy is described as the concurrent use of five or more medications. It is a worrisome public health concern, especially with increasing age and long-term prevalence of chronic diseases [1,2]. In older adults, the risk of polypharmacy is well-founded; its impact on middle-aged individuals (aged 40-59) has drawn far less interest [3]. There is a rise in complicated comorbidities like diabetes, hypertension, cardiovascular diseases, and other complex health conditions in this age group, all of which need multiple drug regimens [4]. Therefore, the combined effect of these drugs, including adverse reactions and overdose risk, has not been well studied in this population.

As there is an exponential rise in prescription drug use across the entire adult population in the United States (US), analyzing certain International Classification of Diseases, Tenth Revision (ICD-10) codes within the national mortality data presents an opportunity to estimate temporal trends to unveil missed public health issues [5].

This study examines trends in drug-related mortality among US adults aged 40-59 between 2018 and 2023 using data from the CDC WONDER (Wide-ranging ONline Data for Epidemiologic Research) Multiple Cause of Death (MCOD) database [6]. By paying attention to the number of deaths involving ICD codes related to adverse effects and poisoning, we are aiming to evaluate whether these patterns can act as a proxy for the growing load of polypharmacy in middle-aged adults. Comprehending these trends can guide public health policies, prospective prescribing operations, and drug safety strategies for high-risk adults below the commonly studied geriatric cutoff.

## Materials and methods

This study was a repeated cross-sectional time trend analysis of drug-related mortality among US adults aged 40-59 between 2018 and 2023, using national mortality data accessed through the CDC WONDER system. Specifically, data were retrieved from MCOD files for the years 2018 through 2023, which are based on death certificates for all US residents and include both the underlying cause of death and additional multiple causes, coded according to the ICD-10.

Deaths were included if the decedent was aged 40-59 years and if any ICD-10 code corresponding to drug poisoning or adverse drug reactions was listed as a contributing cause of death [7]. The study included two groups of ICD-10 codes. The first group, T36-T50, represents poisoning by drugs, medicaments, and biological substances, which includes both intentional and unintentional overdoses, as well as complications related to drug toxicity. The second group, Y40-59, corresponds to adverse drug reactions in therapeutic doses. While these categories capture distinct clinical phenomena, both result in clinically significant drug-related harm. For these reasons, they were combined to provide a comprehensive measure of drug-related mortality, with separate analyses conducted to examine the individual contributions of each category. These ICD-10 codes are publicly available through the US National centre for Health Statistics and do not require a license for research purposes.

Age was categorized into four subgroups for analytical purposes: 40-44 years, 45-49 years, 50-54 years, and 55-59 years, to align with CDC WONDER population estimates and to allow detailed examination of age-specific mortality trends within the midlife adult population. Key variables extracted included year of death, age group, and all MCOD codes. Crude mortality rates per 100,000 population were calculated for each year and age group using the CDC WONDER population estimates as denominators. These rates are not adjusted for demographic differences such as sex, race, or ethnicity, which may influence mortality patterns. Temporal trends in mortality rates were examined to assess changes over time and age-specific differences in mortality burden. No formal statistical tests were performed, as the analysis aimed to provide an overview of population-level trends. All data were exported into Microsoft Excel (Microsoft Corporation, Redmond, Washington, US) for further analysis. This study used publicly available, deidentified data, and, therefore, institutional review board approval was not required for this analysis.

## Results

**Figure 1:**
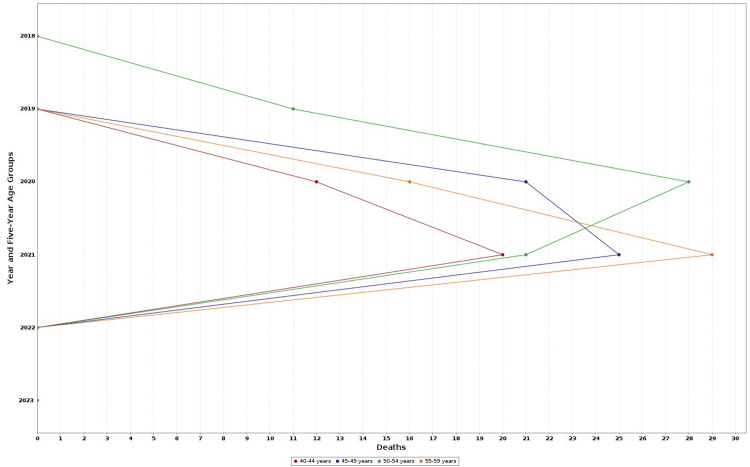
Number of drug-related deaths by year and age group, 2018–2023. This line chart illustrates the trends in total drug-related deaths among adults in the United States aged 40–59 years, stratified by five-year age groups: 40–44, 45–49, 50–54, and 55–59 years, from 2018 to 2023. Data are derived from CDC WONDER multiple cause-of-death files. WONDER: Wide-ranging ONline Data for Epidemiologic Research

**Figure 2:**
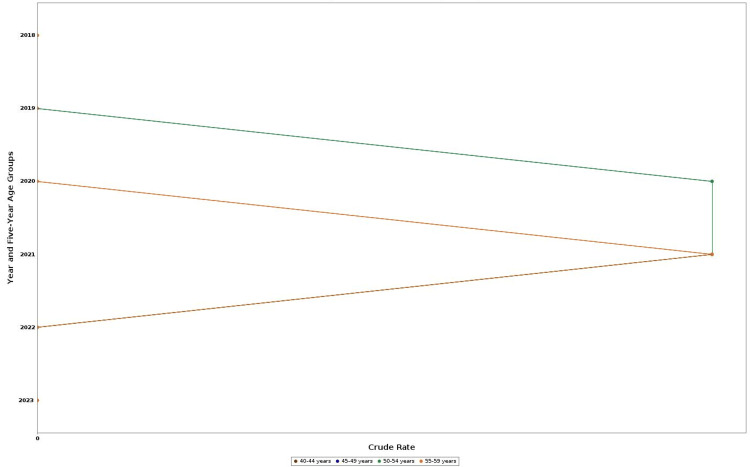
Crude mortality rates by year and age groups, 2018-2023 This line chart shows trends in crude mortality rates (per 100,000 population) among adults in the United States aged 40–59 years, stratified by five-year age groups: 40–44, 45–49, 50–54, and 55–59 years. Data are from CDC WONDER multiple cause-of-death files. Rates are unadjusted for sex, race, or ethnicity. WONDER: Wide-ranging ONline Data for Epidemiologic Research

**Figure 3:**
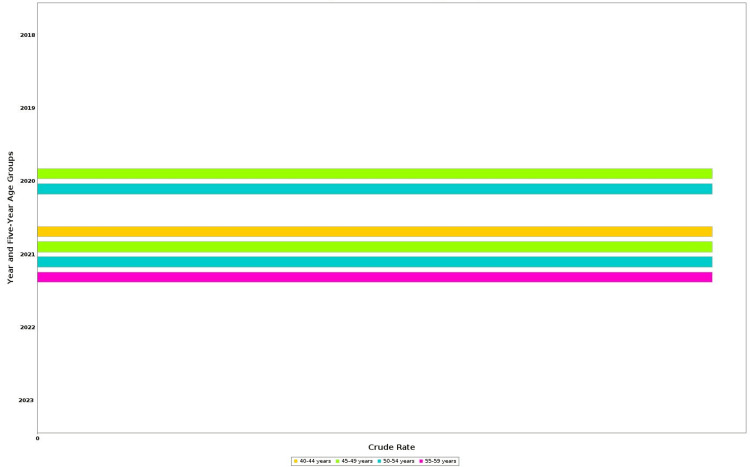
Crude drug-related mortality rates by year and age group, 2018-2023 This bar chart illustrates crude mortality rates (per 100,000 population) among adults in the United States aged 40-59, years.Stratified by age groups: 40–44, 45–49, 50–54, and 55–59 years. Data are from CDC WONDER multiple cause-of-death files. Rates are unadjusted for sex, race, or ethnicity WONDER: Wide-ranging ONline Data for Epidemiologic Research

According to CDC WONDER mortality data, there were 264 drug-related deaths among US adults aged 40-59 years between 2018 and 2023. The total number of deaths rises remarkably from 15 in 2018 to 95 in 2021, followed by a decline to 24 in 2022 and 22 in 2023 (Figures 1, 2). This pattern of mortality correlates with an increase in crude death rate, peaking at 0.1 per 100,000 population in 2020 and 2021 (Figure 3). Given the low absolute rates, observed fluctuations may reflect random variation rather than meaningful changes in mortality patterns. Age-specific analysis showed that the 50-54 and 55- 59 year groups had consistently higher death counts than other groups. Although some information from former years, especially 2018-2019, and some subdivisions were tagged as unreliable due to fewer death counts limiting explainability. Crude mortality rates and total deaths among adults aged 40-59 years increased between 2018 and 2021, followed by a decline in 2022-2023. Age-specific analysis showed higher death counts in the 50-54 and 55-59 year groups compared with younger groups.

## Discussion

Drug-related mortality data indicate an upward trend with age, suggesting an intricate relation among polypharmacy, chronic disease burden, and systemic factors that affect safety regarding medications. The highest crude rates were seen in the 55-59 age group, as the mortality rate increased with age across the full study period (2018-2023) [8,9]. This slope demonstrates a strong age-related susceptibility to mortality, probably affected by complicated treatment plans, medication use, and growing polymorbidities. A noticeable rise was seen from 2020 to 2021 across all groups, followed by a fall in this pattern. This outlines the cumulative effect of the COVID-19 pandemic, including health care services, amplified mental health strain, and expanded opioid usage [10]. Middle-aged adults, a susceptible population due to poorly treated mental health issues, work-related stress, and low utilization of preventive services, are inordinately affected by these changes.

By using drug-related mortality as a proxy for polypharmacy, we feature the public and clinical outcomes of complicated drug regimens in these groups; however, ICD-10 codes also capture overdoses, adverse reactions, and illicit drug use, so not all deaths are attributable to polypharmacy. The upswing trend in mortality, whether from prescribed or self-administered medication, highlights the importance of interventions to prevent deaths in this age group. 

Beyond individual actions, system and structural factors contribute to drug-related mortality. Health disparities, which include limited health care access, under-resourced mental health services, and social factors like housing insecurity, unemployment, and gaps in education, create an environment where risky medical practices are more likely to occur [11]. Furthermore, scattered care systems can also lead to failure of effective communication among health care providers, which further increases the chances of medication error and dangerous drug interactions, most specifically in older patients managing multiple prescriptions at a time [12-14]. These challenges in systems affect vulnerable populations, further increasing the polypharmacy effect and decreasing the effectiveness of preventive interventions [15-17]. During the COVID-19 pandemic, all these problems worsened, polypharmacy became more prevalent, and patients using multiple medications, particularly in hospitals and nursing homes, may have been at higher risk of drug-related mortality. Handling drug-related mortality, therefore, needs a multi-layered method that moves beyond individual-level solutions and involves wider policy changes and health system transformation [18,19]

All these findings need appropriate interventions, including medical reconciliation, optimized screening for substance use disorder, and more robust public health education regarding the risk of polypharmacy [20].

## Conclusions

Drug-related mortality among US adults aged 40-59 increased with age between 2018 and 2022. The rise in the number of deaths from 2020 to 2021, followed by a moderate fall, illustrates the perplexed interaction of polypharmacy, pandemic-related disruptions, and increased substance use. These findings provides preliminary support that drug-related mortality may serve as an impactful proxy for polypharmacy. Advocating this issue requires medication reconciliation strategies, broadening mental health service access, and the development of a national framework to lessen the detrimental outcomes of multiple drug exposure in middle-aged adults .
